# Targeting miR-381-NEFL axis sensitizes glioblastoma cells to temozolomide by regulating stemness factors and multidrug resistance factors

**DOI:** 10.18632/oncotarget.3061

**Published:** 2014-12-18

**Authors:** Zeyou Wang, Jing Yang, Gang Xu, Wei Wang, Changhong Liu, Honghui Yang, Zhibin Yu, Qianqian Lei, Lan Xiao, Jing Xiong, Liang Zeng, Juanjuan Xiang, Jian Ma, Guiyuan Li, Minghua Wu

**Affiliations:** ^1^ Hunan Provincial Tumor Hospital and the Affiliated Tumor Hospital of Xiangya Medical School, Central South University, Changsha, Hunan, China; ^2^ Cancer Research Institute, Central South University, Key Laboratory of Carcinogenesis and Cancer Invasion, Ministry of Education, Key Laboratory of Carcinogenesis, Ministry of Health, Changsha, Hunan, China; ^3^ Department of Ophthalmology, Xiangya Hospital, Central South University, Changsha, Hunan, China

**Keywords:** miRNA, neurofilament light polypeptide, multidrug resistance factor, chemosensitivity, stemness factor

## Abstract

MicroRNA-381 (miR-381) is a highly expressed onco-miRNA that is involved in malignant progression and has been suggested to be a good target for glioblastoma multiforme (GBM) therapy. In this study, we employed two-dimensional fluorescence differential gel electrophoresis (2-D DIGE) and MALDI–TOF/TOF-MS/MS to identify 27 differentially expressed proteins, including the significantly upregulated neurofilament light polypeptide (NEFL), in glioblastoma cells in which miR-381 expression was inhibited. We identified NEFL as a novel target molecule of miR-381 and a tumor suppressor gene. In human astrocytoma clinical specimens, NEFL was downregulated with increased levels of miR-381 expression. Either suppressing miR-381 or enforcing NEFL expression dramatically sensitized glioblastoma cells to temozolomide (TMZ), a promising chemotherapeutic agent for treating GBMs. The mechanism by which these cells were sensitized to TMZ was investigated by inhibiting various multidrug resistance factors (ABCG2, ABCC3, and ABCC5) and stemness factors (ALDH1, CD44, CKIT, KLF4, Nanog, Nestin, and SOX2). Our results further demonstrated that miR-381 overexpression reversed the viability of U251 cells exhibiting NEFL-mediated TMZ sensitivity. In addition, NEFL-siRNA also reversed the proliferation rate of U251 cells exhibiting locked nucleic acid (LNA)-anti-miR-381-mediated TMZ sensitivity. Overall, the miR-381-NEFL axis is important for TMZ resistance in GBM and may potentially serve as a novel therapeutic target for glioma.

## INTRODUCTION

Glioblastoma multiforme (GBM), the most malignant form of glioma, is highly aggressive and neurologically destructive [1, 2]. Despite the combination of surgery, radiotherapy and chemotherapy, the average life expectancy of GBM patients is only 15 months [3]. Although temozolomide (TMZ), a DNA alkylating antineoplastic drug, has been used universally in GBM patients and has been shown to restrain tumor growth for a few months, its resistance is common and accounts for many treatment failures [4, 5]. Recent studies have linked this drug resistance to the altered expression of miRNAs, and therefore, miRNA-based approaches modulating the sensitivity to TMZ could help to overcome chemoresistance [6-11].

It is known that miRNAs play important roles in cancer cell proliferation, aggressiveness and metastasis [12]. It has been reported that miR-195, miR-455-3p and miR-10a* are highly expressed in the induced TMZ-resistant U251R cell line and that miR-195 inhibition enhances TMZ-induced cell death [6]. Additionally, combined treatment of miR-21 inhibitor with TMZ significantly enhances human glioblastoma stem cell apoptosis [7], and miR-143, as a tumor suppressor, sensitizes glioma cells to TMZ by targeting N-RAS [8]. Interestingly, miR-125b inhibitor boosts the chemosensitivity of glioblastoma stem cells to TMZ by targeting Bak1 [9], whereas the miR-128, miR-149 and miR-181 families enhance the chemosensitivity of glioblastoma cells to TMZ by targeting Rap1B [10, 11].

Furthermore, miR-381 is involved in the development and progression of multiple types of cancers, such as prostate cancer [13], renal cancer [14], lung cancer [15], ovarian cancer [16] and glioma [17, 18]. Additionally, miR-381 has been shown to modulate sensitivity to chemotherapeutic agents. For example, miR-381 increases the sensitivity of renal cancer cells to 5-fluorouracil (5-FU) by inhibiting WEE1 [14], and miR-381 modulates the MDR phenotype in leukemia cells and increases their drug uptake [19]. Notably, miR-381 expression is significantly higher in drug-resistant than in drug-sensitive ovarian cancer tissues. Our previous study indicated that miR-381 plays an oncogenic role and is involved in the pathogenesis of GBM [17]. Furthermore, silencing miR-381 inhibits the growth of intracranially transplanted GBM in rats, as determined by magnetic resonance imaging [18]. Here, we explored the effects of miR-381 inhibition on the chemosensitivity of GBM cells to TMZ.

The NEFL gene encodes a type IV intermediate filament, which forms heteropolymers that functionally maintain the neuronal caliber and play an important role in intracellular transport of neurotransmitters to axons and dendrites [20]. NEFL is located on chromosome 8p21, which has been identified as a genetic locus frequently affected by both heterozygous and homozygous deletions in a variety of common human cancers, including prostate cancer [21], breast cancer [22, 23] and head and neck cancer (HNC) [24, 25]. These characteristics implicate NEFL as a potential tumor suppresser gene. NEFL is downregulated by its hypermethylation in breast cancer [23] and HNC [25]. Downregulated NEFL expression due to hypermethylation was associated with resistance to cisplatin-based chemotherapy, and re-expression of NEFL significantly increased the sensitivity of HNC to the drug through the mTOR pathway [25].

Based on research suggesting that miR-381 promotes the growth of glioblastoma cells [17, 18], we performed 2D-DIGE and MALDI-TOF mass spectrometry to screen differentially expressing proteins regulated by miR-381. We identified NEFL as a novel target of miR-381 that was apparently downregulated in GBM, and we showed that inhibition of miR-381 enhanced the sensitivity of NEFL-mediated stemness factors to TMZ in GBM.

## RESULTS

### Differentially expressed proteins regulated by LNA-anti-miR-381

**Figure 1:**
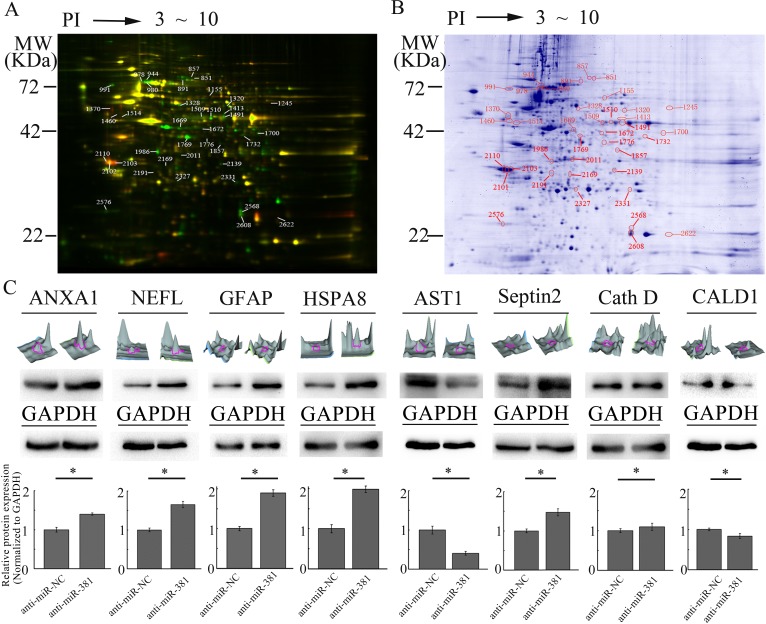
Characterization of different proteins regulated by LNA-anti-miR-381 in GBM cells A: The 2D-DIGE analysis of the different proteins regulated by LNA-anti-miR-381 in GBM cells. U251-LNA-anti-miR-NC and U251-LNA-anti-miR-381 cell lysates as well as internal standard proteins were treated with ReadyPrep 2D reagents, labeled with Cy3, Cy5, or Cy2, respectively, and subjected to 2-D electrophoresis. The data shown here are representative images from three separate experiments. The green spots represent the upregulated proteins; the red spots are the downregulated proteins; and the yellow spots are the internal standard proteins. B: Preparative 2D-PAGE map. The spots accompanied by a number are those resulting from the matching between the preparative and analytical gel protein maps. The protein spots of interest were excised from the gel, digested with trypsin and identified by MALDI-TOF MS peptide mass fingerprinting. The numbers refer to the identified proteins shown in Table 1. C: Analysis of the eight differentially expressed proteins. Top row of images: three-dimensional peak maps of the differentially expressed protein spots. Second row of images: western blotting validation of the differentially expressed proteins, including ANXA1, NEFL, GFAP, HSPA8, Septin 2, Cath D, AST1 and CALD1 (upper panel) and control GAPDH (lower panel). Third row of panels: quantitative analysis of the differentially expressed proteins from three separate experiments. Anti-miR-NC: U251 cells transfected with LNA-anti-miR-NC; and anti-miR-381: U251 cells transfected with LNA-anti-miR-381. The data represent the mean±SDs of 3 replicates. *p <0.05.

After performing 2-D DIGE, the Cy2, Cy3, and Cy5 channels of the individual gels were imaged and analyzed using the DeCyder 5.0 software. As shown in (Fig. 1A), 39 matched protein spots had significant differences in the signal intensity between the U251 cells transfected with LNA-anti-miR-381 or LNA-anti-miR-NC, suggesting that these proteins were differentially expressed. Fifteen of the 39 proteins were significantly upregulated in U251 cells transfected with LNA-anti-miR-381 inhibitor as the ratio of the protein in U251-LNA-anti-miR-381 to that in U251-LNA-anti-miR-NC was ≥ 2 (P ≤ 0.05), while 24 out of 39 proteins were significantly down-regulated (P ≤ 0.05). The 39 protein spots were excised from the gel in (Fig. 1B), digested with trypsin and identified by MALDI-TOF MS peptide mass fingerprinting. Twenty seven differentially expressed proteins were successfully identified by MS (Table 1), and were functionally involved in metabolism, proliferation,signal transduction, structural protein, translation, cell death, autophagic, cytoskeleton organization, chaperone and so on (Table 1), annotation using the Swiss-Prot database).

To verify their differential expression, the U251-LNA-anti-miR-NC and U251-LNA-anti-miR-381 cell lysates were analyzed by immunoblotting using antibodies against annexin I (ANXA1), neurofilament, light polypeptide 68 kDa (NEFL), glial fibrillary acidic protein (GFAP), heat shock 70 kDa protein 8 isoform 1 (HSPA8), aspartate aminotransferase 1 (AST1), Septin 2, Cathepsin D (Cath D) and caldesmon 1 isoform 2 (CALD1). As shown in (Fig. 1C), the relative levels of ANXA1, NEFL, GFAP, HSPA8, Septin 2 and Cath D expression to the levels of the GAPDH control were upregulated in U251-LNA-anti-miR-381-transfected cells, compared to U251-LNA-anti-miR-NC cells, but NEFL expression was particularly upregulated. In contrast, the relative levels of AST1 and CaMBP expression to GAPDH control levels were downregulated in U251-LNA-anti-miR-381 cells. These two independent lines of evidence demonstrated that these proteins were regulated by LNA-anti-miR-381 inhibitor in glioblastoma cells.

**Table 1:** Differentially Expressed Proteins Identified by MALDI TOF/TOF

Spot No.	Accession No.	Protein name	
up-regulation			
980	gi|5729877	Heat shock 70kDa protein 8 isoform 1	Chaperone; Transcription regulation; axon guidance
991	gi|105990539	Neurofilament, light polypeptide 68kDa	Intermediate filament; axon transport; cell death
1155	gi|27436946	Lamin A/C isoform 1 precursor	Intermediate filament; cell migration; establishment or maintenance of microtubule cytoskeleton polarity; cell development
1672	gi|40788885	KIAA0158(Septin-2)	Cell cycle; division;Mitosis; neuron projection development; transport; Signal transduction
1986	gi|4502101	Annexin I	cell differentiation; Signal transduction; inflammatory response; protein secretion; cell cycle; cell proliferation; apoptotic process
2103	gi|19072649	TPMsk3	Structural protein
2110	gi|4507651	Tropomyosin 4	Structural protein; differentiation; cellular component movement; muscle filament sliding
2169	gi|494296	Chain B, Cathepsin D	Cell death; autophagic; collagen catabolic; extracellular matrix disassembly and organization
2327	gi|57997051	Glial fibrillary acidic protein	Intermediate filament; cell differentiation; cell development; extracellular matrix organization; intermediate filament organization; cell proliferation; neurotransmitter uptake
2622	gi|50949396	Holliday junction recognition protein	Chaperone; cell cycle;
			
down-regulation			
851	gi|4826657	Caldesmon 1 isoform 2	Cytoskeleton;cellular component movement; muscle contraction
978	gi|62414289	Vimentin	Intermediate filament; cell differentiation; cell development; apoptotic ; cellular component movement; neuron projection development;
1320	gi|66361514	A Deletion Variant Of Human Glucose 6-Phosphate Dehydrogenase Complexe	Metabolism
1328	gi|860986	Protein disulfide isomerase	Metabolism; signal transduction; Chaperone
1413	gi|4503571	Enolase 1	Metabolism; cell growth; transcription;
1509	gi|693933	2-phosphopyruvate-hydratase alpha-enolase; carbonate dehydratase	Metabolism
1669	gi|15277503	ACTB protein	Structural protein
1700	gi|1526426	Proteasome subunit p42	Metabolism; cell cycle; apoptotic; gene expression;cell cycle
1732	gi|4504067	Aspartate aminotransferase 1	Metabolism
1769	gi|1322019	Uroporphyrinogen decarboxylase	Metabolism
1776	gi|1066756	Beta-globin	Metabolism
1857	gi|67464392	Chain A, Structure Of Human Muscle Pyruvate Kinase	Metabolism
2139	gi|7513022	Hypothetical protein KIAA0567 - human (fragment)	Metabolism
2331	gi|999892	Chain A, Triosephosphate Isomerase Complexed With 2-Phosphoglycolic Acid	Metabolism
2568	gi|388307	Transcription factor	Transcription
2576	gi|4757908	Calcyphosine isoform a	Signal transduction
2608	gi|4503057	Crystallin, alpha B	Aging; apoptotic; microtubule polymerization or depolymerization;

### NEFL is a new target molecule of miR-381

**Figure 2:**
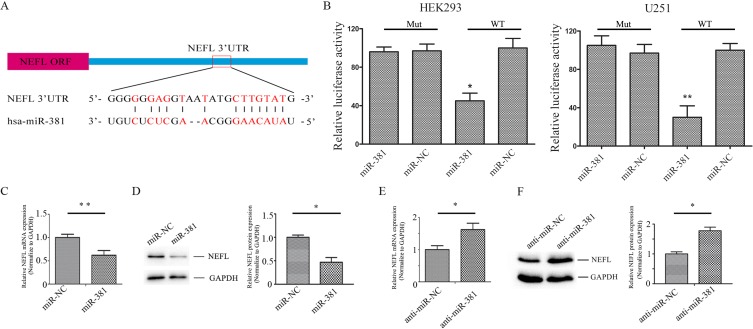
NEFL is a target molecule of miR-381 A: Schematic of the interaction sites of miR-381 in the 3′-UTRs of NEFL. B: Luciferase assays of HEK293 and U251 cells co-transfected with pMIR-REPORT-WT/mutant 3′-UTR NEFL and miR-381 or the negative control, as indicated. C: RT-qPCR analysis showing the mRNA level of NEFL after miR-381 mimics were transfected into U251 cells for 24 h. miR-381 downregulated the mRNA level of NEFL. D: Western blot analysis showing the protein expression of NEFL after miR-381 mimics were transfected into U251 cells for 48 h. miR-381 decreased the protein expression of NEFL; GAPDH was used as a loading control. E: RT-qPCR analysis showing the mRNA level of NEFL after LNA-anti-miR-381 was transfected into U251 cells for 24 h. LNA-anti-miR-381 upregulated the mRNA level of NEFL. F: Western blot analysis showing the protein expression of NEFL after LNA-anti-miR-381 was transfected into U251 cells for 48 h. LNA-anti-miR-381 increased the protein expression of NEFL; GAPDH was used as a loading control. The data represent the mean±SDs of 3 replicates. * *p* <0.05; ** *p* <0.01.

The differentially expressed protein NEFL has been thought to be a putative target of miR-381 by miRanda (Fig. 2A). Therefore, HEK293 and U251 cells were co-transfected with the wild type (WT) or mutated (Mut) NEFL luciferase reporter vector, together with miR-381 or miR-NC, for 24 h. Luciferase activity was significantly reduced in cells transfected with WT NEFL and miR-381, but not in the cells transfected with Mut NEFL and miR-381 (Fig. 2B). Quantitative real time (qRT) PCR and western blotting analysis indicated that the expression of the NEFL mRNA and protein was downregulated in miR-381-treated U251 cells (Fig. 2C and D) but was increased in LNA-anti-miR-381-transfected cells (Fig. 2E and F). These results suggest that miR-381 directly targets NEFL by binding to its seed region in their 3′-UTRs.

### NEFL is reduced in glioma tissues and cell lines, and its overexpression suppresses the proliferation and invasion of U251 cells

**Figure 3:**
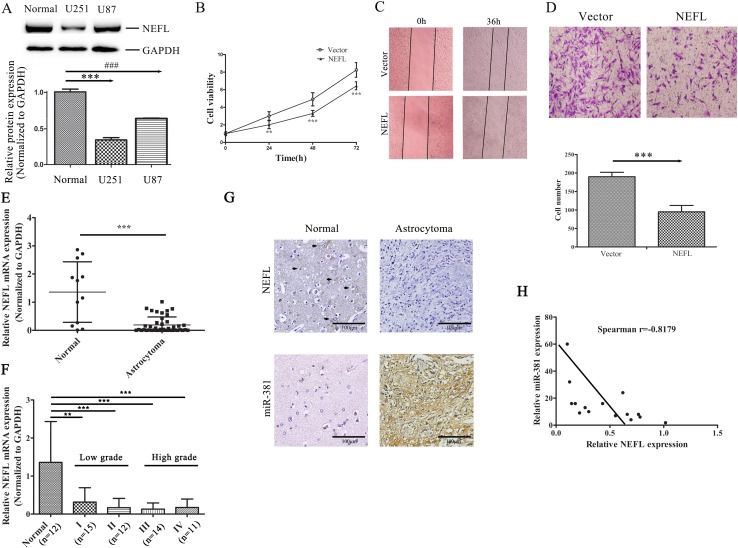
The expression of NEFL is reduced in astrocytomas, and NEFL overexpression suppresses the proliferation and invasion of U251 cells A: Western blot analysis showing that the NEFL protein level was reduced in glioma cells compared to normal brain tissues. Normal: normal brain tissue. B: CCK8 assay showing the reduced proliferation of U251 cells transfected with NEFL. C: NEFL inhibits tumor cell migration, as determined by *in vitro* wound healing assays. D: Matrigel chamber invasion assay showing reduced invasion of U251 cells after being transfected with NEFL. E: RT-qPCR analysis showing that the mRNA level of NEFL was significantly reduced in astrocytoma tissues (n=52) compared to normal brain tissues (n=12). F: RT-qPCR analysis showing that the NEFL expression in high-grade astrocytomas was not different from that in low-grade astrocytomas. G: Immunohistochemistry analysis of NEFL expression (upper) in normal brain tissue and astrocytoma tissue (brown color for positive cells; noted with black arrows). In situ hybridization analysis of miR-381 expression (lower) in normal brain tissue and astrocytoma tissue. H: Spearman's correlation analysis was used to determine the correlation between the expression levels of NEFL and miR-381 in human astrocytomas; Spearman's correlation, r =-0.8179 (n=15). The data represent the mean±SDs of 3 replicates. ** *p* <0.01; *** *p* <0.001.

First, we analyzed the expression of NEFL in the glioblastoma cell lines U251 and U87. We showed that the NEFL protein level was reduced in glioblastoma cells compared to non-tumor brain tissues (Fig. 3A). Interestingly, the expression of NEFL was slightly higher in U87 than U251 cells, and the U87 cells were less resistant to TMZ than the U251 cells (Fig. S1C). Compared with cells transfected with the empty vector, NEFL overexpression inhibited the proliferation (Fig. 3B), migration (Fig. 3C), and invasion (Fig. 3D) of the U251 cells. We also used U87 cells to assess the role of NEFL in regulating the cell proliferation (Fig. S1A) and invasion (Fig. S1B) of GBM cells, and these results were consistent with those of the U251 cells.

Our previous study showed that relative to normal adult brain, miR-381 was highly expressed in different brain cancer subtypes, including GBM[17]. Therefore, we measured the expression levels of NEFL in 12 normal brain tissues and 52 astrocytoma samples. RT-qPCR assays showed that NEFL expression was significantly decreased in the astrocytoma samples compared with the normal brain tissues (Fig. 3E). We then divided the astrocytoma samples into grade I (n=15), grade II (n=12), grade III (n=14), or grade IV (n=11) according to WHO classification. NEFL was downregulated in the 4 astrocytoma groups compared with the normal brain group (*p*≤0.01; Fig. 3F). However, the NEFL expression in high-grade tumors (WHO Grades III and IV) showed no significant differences compared to that of low-grade tumors (WHO Grade I and II) (Fig. 3F). Immunohistochemistry analysis of the NEFL expression in normal brain and astrocytoma tissues showed a similar result (Fig. 3G). Moreover, we analyzed the expression pattern of miR-381 in tissues using in situ hybridization (Fig. 3G). Because NEFL is a target molecule of miR-381, we also determined the correlation between NEFL and miR-381 in astrocytoma samples. We observed that NEFL was inversely correlated with miR-381 expression in the analyzed astrocytomas (n=15) (Spearman's correlation, r = −0.8179) (Fig. 3H), which suggested that NEFL acts as a putative tumor suppressor in glioma and that both downregulated NEFL and upregulated miR-381 expression are involved in gliomagenesis.

### NEFL increases the chemosensitivity of glioblastoma cells to TMZ by regulating multidrug resistance and stemness factors

**Figure 4:**
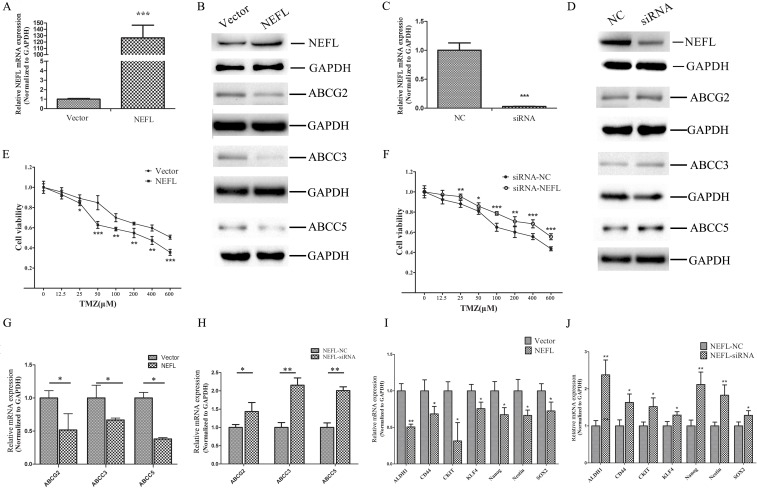
NEFL affects the chemosensitivity of glioblastoma cells to TMZ by regulating multidrug resistance and stemness factors A: RT-qPCR analysis showing that the NEFL protein level is significantly increased in U251 cells upon NEFL overexpression. B: Western blot analysis showing that the protein expression of NEFL is upregulated in U251 cells and that this overexpression leads to the downregulated expression of the ABCG2, ABCC3 and ABCC5 proteins in U251 cells. C: U251 cells transfected with empty or NEFL vector were treated with various concentrations of TMZ for 48 h and then subjected to CCK8 assays. NEFL overexpression significantly increased the chemosensitivity of U251 cells to TMZ treatment. D: RT-qPCR analysis showing that the expression of multidrug resistance factors (ABCG2, ABCC3, ABCC5) was decreased in U251 cells overexpressing NEFL. E: RT-qPCR analysis showing that the expression of stemness factors (ALDH1, CD44, CKIT, KLF4, Nanog, Nestin, SOX2) was downregulated in U251 cells overexpressing NEFL. F: RT-qPCR analysis showing that the NEFL protein level was significantly decreased in U251 cells after treatment with NEFL-siRNA. G: Western blot analysis showing that the protein expression of NEFL was reduced after treatment with NEFL-siRNA and that the protein expression of ABCG2, ABCC3 and ABCC5 was simultaneously upregulated in U251 cells. H: U251 cells transfected with siRNA-NC or siRNA-NEFL were treated with various concentrations of TMZ for 48 h and then subjected to CCK8 assays. Knockdown of NEFL decreased the chemosensitivity of the cells to TMZ treatment. I: RT-qPCR analysis showing that the expression of multidrug resistance factors (ABCG2, ABCC3, ABCC5) was increased in U251 cells treated with NEFL-siRNA. J: RT-qPCR analysis showing that the expression of stemness factors (ALDH1, CD44, CKIT, KLF4, Nanog, Nestin, SOX2) was upregulated in U251 cells treated with NEFL-siRNA. The data represent the mean±SDs of 3 replicates. * *p*<0.05; ** *p* <0.01; *** *p* <0.001.

Resistance to TMZ is one of the major causes of failed GBM chemotherapy; therefore, it is critical to discover new strategies that increase the effectiveness of TMZ treatment. After NEFL overexpression (Fig. 4A and B) or NEFL knockdown (Fig. 4C and D), we treated U251 cells with different concentrations of TMZ. As shown in (Fig. 4E), when compared to control group cells, NEFL overexpression significantly increased the chemosensitivity of U251 cells to TMZ treatment, as observed by the significant suppression of cell viability after TMZ treatment and by the inverse correlation of the drug concentrations to cell viability. However, NEFL siRNA decreased the chemosensitivity of the cells to TMZ treatment (Fig. 4F). To identify the mechanism by which NEFL enhances the chemosensitivity of glioblastoma cells to TMZ, the expression of multidrug resistance factors was analyzed by RT-qPCR. NEFL overexpression significantly downregulated the multidrug resistance factors ABCG2, ABCC3, and ABCC5 in U251 cells, both at the protein (Fig. 4B) and mRNA level (Fig. 4G). Similar results were obtained for U87 cells (Fig. S1E). Furthermore, the expression of ABCG2, ABCC3, and ABCC5 was upregulated after inhibiting the expression of NEFL (Fig. 4D and H).

We also analyzed the expression of the stemness factors ALDH1, CD44, CKIT, KLF4, Nanog, Nestin, and SOX2. NEFL overexpression decreased the expression of ALDH1, CD44, CKIT, KLF4, Nanog, Nestin, and SOX2 (Fig. 4I), whereas silencing NEFL increased the expression of ALDH1, CD44, CKIT, KLF4, Nanog, Nestin, and SOX2 (Fig. 4J). The above-mentioned data indicate that NEFL increases the chemosensitivity of glioblastoma cells to TMZ by regulating multidrug resistance and stemness factors. We observed similar results in U87 cells (Fig. S1E).

### Targeted inhibition of miR-381 increases the sensitivity of glioblastoma cells to TMZ by upregulating NEFL expression

**Figure 5:**
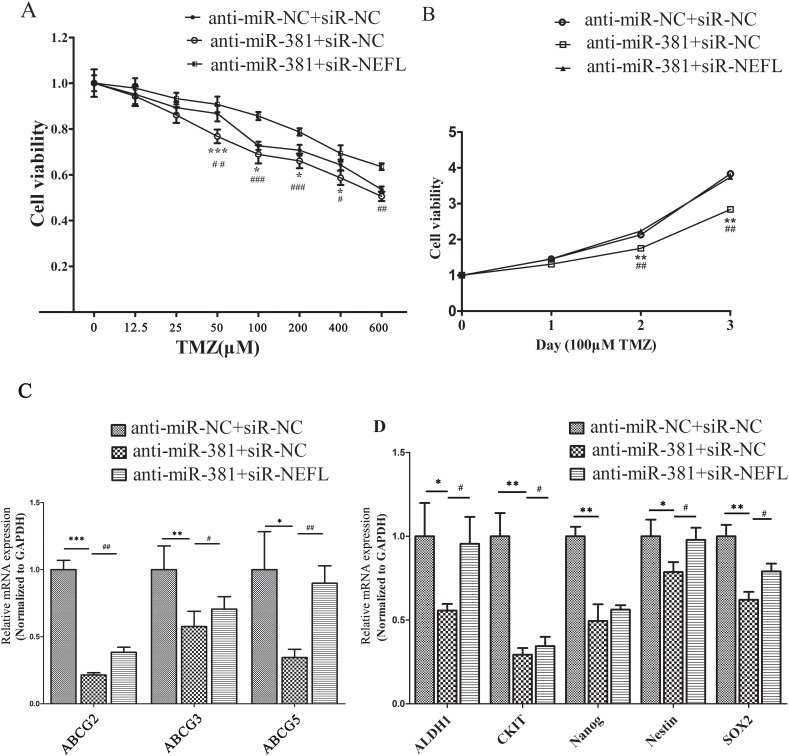
NEFL-siRNA reverses the sensitivity of LNA-anti-miR-381-treated cells to TMZ A: U251 cells transfected with LNA-anti-miR-NC or LNA-anti-miR-381 were forced to repress the expression of NEFL, were treated with various concentrations of TMZ for 48 h and were then submitted to CCK8 assays. The results showed that LNA-anti-miR-381 significantly increased the chemosensitivity of U251 cells to TMZ. Silencing the expression of NEFL inhibited the effects of LNA-anti-miR-381. B: U251 cells transfected with LNA-anti-miR-NC or LNA-anti-miR-381 were forced to repress the expression of NEFL, were treated with 100 μM TMZ for the indicated times, and were then subjected to CCK8 assays. The results showed that transfected LNA-anti-miR-381 decreased the proliferation of U251 cells and enhanced their sensitivity to TMZ. NEFL-siRNA prevented the sensitizing effects of LNA-anti-miR-381 to TMZ. C: RT-qPCR analysis showing that the mRNA level of multidrug resistance factors (ABCG2, ABCC3, ABCC5) were repressed in U251 cells after LNA-anti-miR-381 treatment and restored by transfection of the NEFL siRNA. D: RT-qPCR analysis showing that the mRNA level of stemness factors (ALDH1, CKIT, Nanog, Nestin, SOX2) was repressed in U251 cells after LNA-anti-miR-381 treatment and was restored by transfection of the NEFL siRNA. The data represent the mean±SDs of 3 replicates. *Indicates a significant difference compared to the LNA-anti-miR-NC+siR-NC group, #indicates a significant difference compared to the LNA-anti-miR-381+siR-NEFL group. * *p* <0.05; ** *p* <0.01; *** *p* <0.001; # *p* <0.05; ## *p* <0.01; ### *p* <0.001.

First, we examined the effects of targeted inhibition of miR-381 on the increased sensitivity of glioblastoma cells to TMZ. As expected, suppression of miR-381 by LNA-anti-miR-381 significantly increased the chemosensitivity of U251 cells to TMZ treatment (*, *p* <0.05) (Fig. 5A). Next, we investigated whether NEFL expression is critical for the LNA-anti-miR-381-mediated cellular sensitivity of U251 cells to TMZ. After the cells were transfected with LNA-anti-miR-NC or LNA-anti-miR-381, they were transfected with NEFL siRNAs for 8 h and then treated with different concentrations of TMZ. Silencing the expression of NEFL inhibited the effects of LNA-anti-miR-381 chemosensitivity enhancement (#, *p* <0.05) (Fig. 5A). Moreover, a CCK8 proliferation assay was used to explore the role of NEFL in LNA-anti-miR-381-mediated cell proliferation in the presence of TMZ (100 μM), and cell viability was determined at the indicated time points. We found that inhibition of miR-381 decreased the proliferation and enhanced the sensitivity of U251 cells to TMZ (Fig. 5B), while silencing the expression of NEFL with siRNA resisted the sensitizing effects of LNA-anti-miR-381 to TMZ (Fig. 5B). Subsequently, the multidrug resistance and stemness factors were also investigated using RT-qPCR. LNA-anti-miR-381 downregulated the expression of multidrug resistance factors (ABCG2, ABCC3, and ABCC5) and stemness factors (CD44, CKIT, KLF4, Nanog, and Nestin2) in these cells (Fig. 5C and D). Interestingly, when NEFL expression was interfered with during the LNA-anti-miR-381 treatment, the expression of the multidrug resistance factors (ABCG2, ABCC3, and ABCC5) and stemness factors (CD44, CKIT, KLF4, Nanog, and Nestin2) (Fig. 5C and D) was restored. We observed similar results in U87 cells (Fig. S1F). These results suggested that NEFL siRNA reverses the proliferation rate of LNA-anti-miR-381-transfected, TMZ-sensitive U251 cells. To our knowledge, this is the first report to show that miR-381 regulates the chemosensitivity of glioblastoma cells to TMZ treatment through expressing NEFL.

### miR-381 mimics disrupt the sensitization of NEFL to TMZ in glioblastoma cells

**Figure 6:**
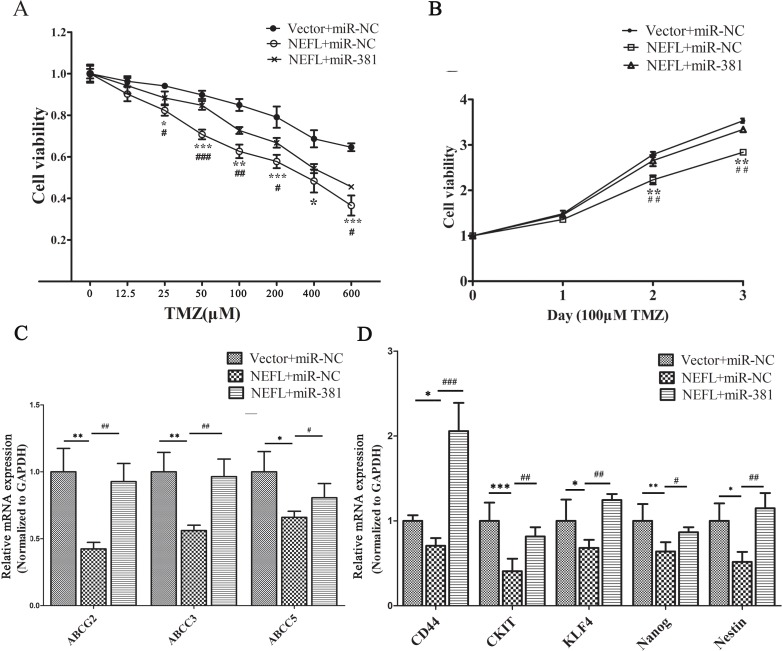
miR-381 disrupts the sensitization of U251 cells to TMZ via NEFL A: U251 cells were transfected with either empty vector or NEFL and were forced to overexpress miR-381. Subsequently, the cells were treated with various concentrations of TMZ for 48 h and then submitted to CCK8 assays. The results showed that NEFL overexpression significantly increased the chemosensitivity of U251 cells to TMZ treatment. miR-381 inhibited the effects of NEFL. B: U251 cells were transfected with either empty vector or NEFL and were forced to overexpress miR-381. The cells were then treated with 100 μM TMZ for the indicated times and then subjected to CCK8 assays. The results showed that NEFL decreased the proliferation and enhanced the sensitivity of U251 cells to TMZ, while miR-381 inhibited the sensitizing effects of NEFL to TMZ. C: RT-qPCR analysis showing that the mRNA levels of multidrug resistance factors (ABCG2, ABCC3, ABCC5) were repressed in U251 cells after NEFL overexpression and were restored by miR-381. D: RT-qPCR analysis showing that the mRNA level of stemness factors (CD44, CKIT, KLF4, Nanog, and Nestin) were repressed in U251 cells after NEFL overexpression and were restored by miR-381. The data represent the mean±SDs of 3 replicates. *Indicates a significant difference compared to the Vector+miR-NC group, #indicates a significant difference compared to the NEFL+miR-381 group. * *p* <0.05; ** *p* <0.01; *** *p* <0.001; # *p* <0.05; ## *p* <0.01; ### *p* <0.001.

We then investigated whether miR-381 is involved in the NEFL-mediated increased sensitivity of glioblastoma U251 cells to TMZ by transfecting the cells with miR-381 mimics (pre-transfected with vector or NEFL) for 8 h and then treating the cells with different concentrations of TMZ. Cell viability was then determined after 48 h of treatment. As shown in (Fig. 6A), the overexpression of NEFL in U251 cells significantly increased their chemosensitivity to TMZ treatment, whereas miR-381 overexpression inhibited their chemosensitivity. Furthermore, cell viability in the presence of TMZ (100 μM) was assayed at different time points. NEFL overexpression significantly prevented the proliferation of U251 cells in the presence of TMZ; however, this inhibited proliferation was restored by miR-381 overexpression (Fig. 6B). Interestingly, miR-381 overexpression increased the expression of multidrug resistance factors and stemness factors that were blocked by NEFL overexpression combined with TMZ treatment (Fig. 6C and 6D). These results suggest that NEFL induces chemosensitivity of U251 cells to TMZ treatment in an miR-381-dependent manner.

### NEFL sensitizes glioblastoma cells to TMZ by inhibiting the mTOR pathway

TMZ can induce the activation of AMPK in glioblastoma cells, while the activation of AMPK inhibits mTOR complex 1 (mTORC1) signaling [26]. Tuberous sclerosis complex 1 TSC1 functions as a molecular inhibitor of the mTOR oncogenic pathway [27], and NEFL has been shown to bind TSC1 and stabilize the TSC1/2 complex [28]. Furthermore, the downregulation of NEFL has been shown to lead to abnormal activation of the mTOR pathway [25]. Therefore, we hypothesized that the abnormal expression of NEFL leading to mTOR pathway change would confer TMZ resistance.

**Figure 7:**
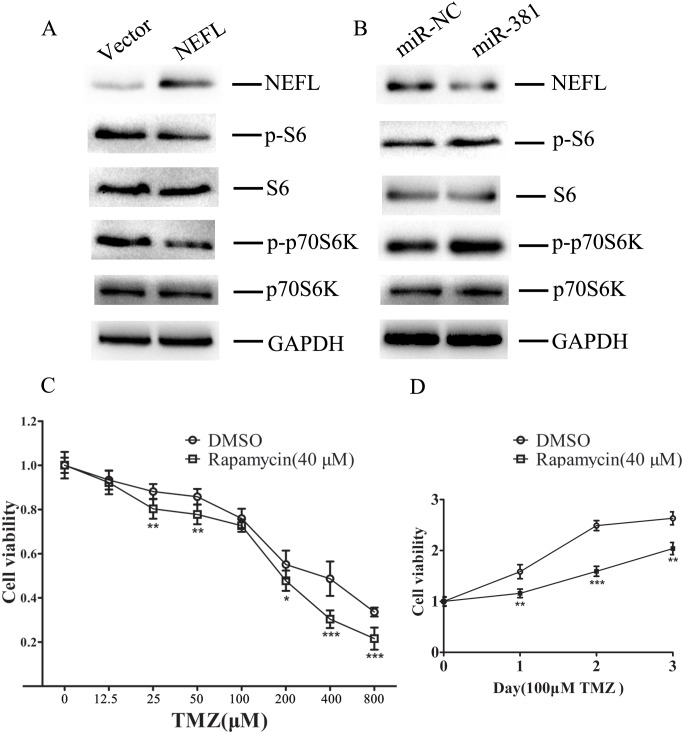
NEFL sensitizes glioblastoma cells to TMZ by inhibiting the mTOR pathway A Western blot analysis showing that the protein expression of NEFL was upregulated in U251 cells after NEFL was overexpressed, whereas the expression of phosphorylated ribosomal protein S6 and p70S6k was downregulated. The expression of total ribosomal protein S6 and p70S6k was not changed. B. Western blot analysis showing that the protein expression of NEFL was downregulated in U251 cells after miR-381 was overexpressed, whereas the expression of phosphorylated ribosomal protein S6 and p70S6k was upregulated. The expression of total ribosomal protein S6 and p70S6k was not changed. C. U251 cells were treated with 40 μM rapamycin for 24 h and then incubated with different concentrations of TMZ and CCK8 assays were conducted 24 h later. Rapamycin treatment increased the chemosensitivity of U251 cells to TMZ. D. U251 cells were treated with 40 μM rapamycin for 24 h and then incubated with TMZ (100 μM), and CCK8 assays were later conducted at different times. Combined treatment of TMZ and rapamycin significantly inhibited U251 cell growth. The data represent the mean±SDs of 3 replicates. * *p*<0.05; ** *p* <0.01; *** *p* <0.001.

To test this hypothesis, we examined the status of the mTOR pathway and the effects of altered NEFL expression in U251 cell lines overexpressing NEFL (Fig. 7A) or miR-381 (Fig. 7B). Consistent with our hypothesis, mTOR pathway activity was inhibited upon NEFL overexpression. The phosphorylation of ribosomal protein S6 and p70S6k, critical downstream substrates of activated mTOR and known indicators of an active mTOR pathway, was then analyzed in U251 cells (Fig. 7A and B). Restoration of NEFL expression using a pcDNA3.1/NEFL vector suppressed p70S6k phosphorylation in U251 cells (Fig. 7A), whereas downregulation of NEFL expression by miR-381 resulted in increased phosphorylation of ribosomal protein S6 and p70S6k in U251 cells (Fig. 7B).

NEFL modulation of mTOR pathway activation indicated a role for mTOR activity in cellular TMZ responsiveness. To test this possibility, we examined the effects of an mTOR inhibitor, rapamycin, on U251 cells. The cells were treated with 40 μM rapamycin for 24 h, incubated with different concentrations of TMZ, and then submitted to CCK8 assays 24 h later. The rapamycin treatment increased the chemosensitivity of the U251 cells to TMZ (Fig. 7C), and most importantly, the combined treatment of TMZ and rapamycin significantly inhibited U251 cell growth (Fig. 7D).

## DISCUSSION

miR-381 has been reported to be an onco-miRNA and to be involved in the tumorigenesis and progression of many cancers [13-18]. Our previous research indicated that miR-381 is highly expressed in different grades of astrocytomas. We also showed that miR-381 targets the LRRC4 gene, a known tumor suppressor of glioma, that overexpression of LRRC4 downregulates the expression of miR-381, and that the interaction between miR-381 and LRRC4 is involved in glioma growth [17]. LNA-anti-miR-381 oligonucleotides inhibited the proliferation of glioblastoma cells *in vitro* and the growth of intracranial transplanted glioblastoma model *in vivo*, as determined by magnetic resonance imaging [18], which suggests that miR-381 is a good target for glioma therapy.

In this study, we analyzed the effects of miR-381 on the proteomic profiles of glioblastoma cells using the 2D-DIGE method. Compared with control cells, 39 proteins were differentially expressed in the LNA-anti-miR-381-treated U251 cells. Twenty-seven proteins, identified by MADLE-TOF-MS/MS, were functionally involved in processes such as metabolism, proliferation, signal transduction, cell structure, translation, cell death, autophagy, inflammation, and cytoskeleton organization, among others, and some were and chaperones. Western blotting analysis indicated that ANXA1, NEFL, GFAP, HSPA8, Septin 2 and Cath D were upregulated, while AST1 and CALD1 were downregulated in LNA-anti-miR-381-treated U251 cells.

Annexin I, which belongs to a family of Ca(2+)-dependent phospholipid-binding proteins, is an important anti-inflammatory factor [29]. NEFL, a type IV intermediate filament, comprises the axoskeleton and plays a key role in intracellular transport to axons and dendrites [20]. GFAP, one of the major intermediate filament proteins of mature astrocytes, is used as a marker to distinguish astrocytes from other glial cells during development [30]. HSPA8, a member of the heat shock protein 70 family, functions as a chaperone and binds to nascent polypeptides to facilitate their correct folding [31]. Septin 2, a conserved family of polymerizing guanosine triphosphate-binding protein, localizes to the metaphase plate during mitosis and is crucial for chromosome congression and segregation [32]. Cath D, a lysosomal aspartyl protease, is associated with autophagy, collagen catabolism, extracellular matrix disassembly and organization [33]. AST1 is a pyridoxal phosphate-dependent enzyme that plays a role in amino acid metabolism and in the urea and tricarboxylic acid cycles [34]. CALD1, a calmodulin- and actin-binding protein, is a potent inhibitor of actin-tropomyosin-activated myosin, MgATPase, and modulates Ca(2+)-dependent inhibition of smooth muscle contraction [35]. We observed that miR-381 affected the metabolism, proliferation, and signal transduction of glioma cells through regulating these proteins. Considering that NEFL is a direct target of miR-381, we focused on the relationship between LNA-anti-miR-381, NEFL, and TMZ chemosensitivity.

GBM is the most aggressive and deadly form of glioma, and TMZ is a promising chemotherapeutic agent for these types of cancer; however, resistance develops quickly and at a high frequency [4, 5]. Several studies have suggested that miRNAs are novel players in the development of chemoresistance. Ectopically expressed miR-34a sensitizes colorectal cancer cells to 5-FU [36], and miR-211 in combination with ionizing radiation (IR) and TMZ treatment significantly induces glioma cell apoptosis and DNA fragmentation [37]. In this study, we found that targeted inhibition of miR-381 promotes the effects of TMZ and that transfection of LNA-anti-miR-381 in combination with TMZ treatment more potently inhibits cell proliferation compared to TMZ treatment alone. Thus, it is important to note that targeted inhibition of miR-381 offers a new modulation strategy to overcome chemoresistance of glioma to TMZ treatment.

NEFL, a potential tumor suppresser [21, 22, 24], is associated with resistance to cisplatin-based chemotherapy, and re-expression of NEFL in HNC significantly increases the sensitivity of the cells to the drug[25]. Our study indicated that NEFL is a new target molecule of miR-381 and is downregulated in astrocytoma. Overexpression of NEFL significantly suppressed the proliferation and invasion of U251 cells and enhanced the chemosensitivity of glioblastoma cells to TMZ. More importantly, the inhibition of NEFL expression by siRNA recovered TMZ resistance after upregulating NEFL expression, which induced TMZ sensitivity, by transfection of an miR-381 inhibitor. Correspondingly, overexpression of miR-381 disrupted the sensitization of glioblastoma cells to TMZ. Thus, the miR-381-NEFL axis is critical for TMZ resistance in GBM, and targeted inhibition of miR-381 or NEFL restoration may offer a new strategy to overcome chemoresistance of glioblastoma to TMZ treatment.

Cancer stem cells are a small population of cells within a tumor that tend to share some common features with stem or progenitor cells, including self-renewal and differentiation capability [38]. These cancer stem cells (CSCs) are thought to be responsible not only for primary tumorigenesis but also for resistance to chemotherapy and subsequent cancer recurrence. Accumulating evidence has suggested that chemotherapy failure might be blamed for the existence of CSCs [39], and a variety of mechanisms have been proposed to contribute to CSC chemoresistance, including relative quiescence, expression of ATP-binding cassette (ABC) transporters and/or multidrug resistance transporter 1 (MDR1), a more robust DNA repair capability, and the elevated expression of antiapoptotic proteins [38]. All of these characteristics together make CSCs a particularly challenging target for chemotherapy [40]. ALDH1, CD44, CKIT, KLF4, Nanog, Nestin, and SOX2 are important stem cell markers. ALDH1, as a novel stem cell marker in human GBM-positive glioblastoma cells, is involved in giving brain tumors stem cell capacity [41]. CD44, a glycoprotein transmembrane receptor, is a marker of stem cells from a variety of normal and neoplastic tissues and is associated with treatment resistance of glioma [42]. The interaction between hyaluronan and CD44 activates the stem cell marker Nanog, Stat-3-mediated MDR1 gene expression, and ankyrin-regulated multidrug efflux in breast and ovarian tumor cells [43], and it can also lead to Bcl-2 expression and chemoresistance in breast cancer cells [44]. CD44+/CD24-/Low CSCs display resistance to conventional chemotherapy. Interestingly, inhibition of Cdk2 kinase activity selectively targets and restores the chemosensitivity of SUM149PT in a CD44+/CD24-/Low stem-like subpopulation of triple-negative breast cancer cells [45]. CKIT is a type 3 transmembrane receptor for mast cell growth factor (MGF) and is a known stem cell factor. Secondary c-Kit mutations confer acquired resistance to RTK inhibitors in c-Kit mutant melanoma cells [46], and KIT copy number gain may be a mechanism by which melanomas acquire therapeutic resistance to imatinib [47]. In addition, promoter hypermethylation of KLF4 inhibits the chemosensitivity of cervical carcinoma to cisplatin [48]. Nanog expression has been found in multipotent brain tumor stem cells (BTSCs) and found to vary from early generation to late generation-BTSCs [49]. TALEN-mediated Nanog disruption results in less invasiveness, more chemosensitivity and reversal of EMT in HeLa cells [50], and knockdown of Nanog enhances the chemosensitivity of liver cancer cells to doxorubicin by reducing MDR1 expression [51]. Nestin is expressed primarily in nerve cells, might contribute to the initiation, promotion, and progression of tumors and is associated with chemotherapy resistance [52,53]. SOX2 is required for stem-cell maintenance in the central nervous system, and Sox2-dependent activation of Wnt signaling drives the development of tamoxifen resistance in cancer stem/progenitor cells [54].

Our research indicated that either suppressing miR-381 or enhancing NEFL expression sensitizes glioblastoma cells to TMZ by inhibiting stemness factors (ALDH1, CD44, CKIT, KLF4, Nanog, Nestin, and SOX2). Our data suggested that targeted inhibition of miR-381 enhances the sensitivity of cells to TMZ in glioblastoma by inhibiting stemness factors and that NEFL regulates the expression of stemness factors.

CD133 is recognized as an important marker to identify and isolate CSCs [55]. Many studies have shown that CD133+ cells are highly chemoresistant [56,57], for example, those in small cell lung cancer [56]. CD133+ melanoma stem-like cells confer resistance to taxol-induced apoptosis [57]; however, Jian Wang *et al*. showed the negative expression of CD133 in U251 and U87 cells [58]. We analyzed the expression of CD133 using real-time qPCR, and the results showed that the expression level of CD133 in U251 cells is very low (data not shown).

Cancer stem cells are recognized to be the origin of cancer and the basis of cancer malignant phenotypes, including multidrug resistance [38]. The multidrug resistance factors ABCG2, ABCC3, and ABCC5 belong to the superfamily of ATP-binding cassette (ABC) transporters [59], which function as xenobiotic transporters and may play a major role in multidrug resistance [60]. Downregulation of ABCG2 expression decreases the chemoresistance of glioblastoma cancer stem cells [61], and ABCC3 and ABCC5 have been reported to be expressed higher than the other ABCC subfamily members in glioma [62, 63]. Furthermore, high expression of ABCC3 and ABCC5 in tumor biopsy samples is linked to a higher risk of death [63, 64]. ABCC3 is expressed more in differentiated glioma cells and regulates multidrug resistance [65, 66], while ABCB1 and ABCC4 are important multidrug resistance factors. Drug-resistant CD133+ CSCs isolated from U138MG cells exhibit increased expression of ABCB1 and ABCC4 [67]; therefore, because the U251 and U87 cell lines are CD133-, we did not focus on ABCB1 and ABCC4.

Our data demonstrated that either suppressing miR-381 or enforcing NEFL expression inhibited the expression of multidrug resistance factors (ABCG2, ABCC3, and ABCC5) in glioblastoma cells in the presence of TMZ. These data suggested that targeted inhibition of miR-381 enhances the sensitivity of glioblastoma cells to TMZ by inhibiting NEFL-mediated expression of stemness and multidrug resistance factors.

In summary, our present research shows that miR-381 is a good target for glioma therapy, and that targeted inhibition of miR-381 enhances the sensitivity of GBM to temozolomide through the regulation of stemness factors by NEFL. Moreover, the miR-381-NEFL axis is critical for TMZ resistance in GBM and may potentially serve as a novel therapeutic target for glioma.

## MATERIALS AND METHODS

### Human tissue samples

Human astrocytoma samples and normal brain tissues were obtained from the Department of Neurosurgery, Xiangya Hospital, Hunan, China. This study was approved by the hospital institutional review board and written informed consent was obtained from all patients. All the protocols were reviewed by the Joint Ethics Committee of the Central South University Health Authority and performed following national guidelines. Tissue samples were collected at surgery, immediately frozen in liquid nitrogen and stored until total RNAs or proteins were extracted.

### Cell culture and reagents

Human glioblatoma cell lines U251 and Human Embryonic Kidney (HEK) 293 cells were maintained in DMEM medium with high glucose and sodium pyruvate, supplemented with 10% fetal bovine serum and antibiotics (100 units/ml penicillin and 100 mg/ml streptomycin). Cells were incubated at 37 °C in a humidified atmosphere of 5% CO2 in air. Antibodies against ANAX1 (610066) was purchased from BD Biosciences (BD Biosciences PharMingen, San Jose, CA). Antibodies against GFAP (#3670), HSPA8 (#8444), p70S6K (#9202) and phospho-p70S6K (#9204) were purchased from Cell Signaling Technology (Beverly, MA, USA). Antibodies against NEFL (1815-1), Cath D (2487-1) and CALD 1 (1089-1) were purchased from Epitomics Inc (Burlingame, CA, USA). Antibodies against AST1 (H00002805-D01) was from Abnova Corporation (Taiwan, China), and antibodies against Ribosomal Protein S6 (sc-74576), phosphor-S6 (sc-54279), Septin 2 (sc-20408) and GAPDH (sc-32233) were from Santa Cruz Biotechnology (Santa Cruz, CA, USA), respectively. Antibodies against ABCG2 (BM0099) was from Abzoom Biolabs, Inc (Dallas, TX,, USA). Antibodies against ABCC3 (DR0076) and ABCC5 (DR5191) was from UcallM Biotechnology Co., Ltd (Wuxi, China).

### Two dimensional differential gel electrophoresis (2-D DIGE), in-gel digestion, and protein identification

The LNA-anti-miR-381 induced differential expression of proteins in glioma cells was characterized by 2-D DIGE analysis, as described previously [68]. Briefly, U251 cells were transfected with LNA-anti-miR-381 or LNA-anti-miR-NC as control. The U251-anti-miR-NC and U251-anti-miR-381 cells were harvested, and the proteins in cell lysates were extracted. Subsequently, these lysate proteins were treated using the ReadyPrep 2D Clean-up kit, according the manufacturers' instruction (Bio-Rad). The lysate proteins were re-suspended in lysis buffer (8 M urea, 4% w/v CHAPS, 30 mM Tris-Cl, pH 8.5), and determined for their protein concentrations using BCA (Pirece, USA). These proteins were labeled with 400 pmol of fluorescence dye per 100m g of lysates proteins using the DIGE labeling solution (GE Healthcare). A total of 20 mg of proteins from each group was mixed with the same volume of DIGE 2 × buffer (8 M urea, 4% w/v CHAPS, 2% w/v DTT, 2% v/v Pharmalytes 3–10 for IEF), and 20 mg of individual samples were diluted in rehydration solution (8 M urea, 0.5% w/v CHAPS, 0.2% w/v DTT, and 0.2% v/v Pharmalyte pH 3-10) and loaded on IPG strips (18 cm, pH 3–10, non-linear, GE Healthcare) for 2-D gel electrophoresis. Fluorescence images were acquired using the Ettan DIGE imager (GE Healthcare), and the DIGE gels were analyzed using the DIA module of the Decyder software (Version 6.5, GE healthcare). To prepare gels for capturing the spots of interest, 500~1000 mg of proteins were subjected to 2-D DIGE on IPG strips and stained with Coomassie Brilliant Blue. The protein spots of interest were excised and destained with 25 mM ammonium bicarbonate/50% acetonitrile (CAN), followed by in-gel digestion with 0.01 mg/ ml trypsin (Promega, USA) in 25 mM ammonium bicarbonate for 15 h at 37°C. The hydrolysates were collected, and the tryptic peptides were extracted from the gel pieces sequentially with 5% TFA at 40°C for 1 h, and with 2.5% TFA, 50% ACN at 30°C for 1 h. The extracts were pooled, lyophilized, and stored at −20°C until use. Gel pieces from a “blank” region and from BSA molecular mass marker were used as negative and positive controls, respectively. The peptide mixtures were re-dissolved in 0.5% of TFA, and 1 ml of peptide solution was mixed with equal volume of matrix (4-hydroxy-alpha-cyanocinnamic acid, HCCA in 30% ACN/0.1% TFA), followed by spotting on the target plate. Individual protein peptides were identified by MALDI-TOF mass spectrometry on a 4700 proteomics analyzer (Applied Biosystems, Foster City, CA). Mass spectrum was used to interrogate human protein sequences in the SWISS-PROT database using the MASCOT database search algo-rithms (version 1.9).

### Quantitative real time-PCR analysis

Real-time PCR was carried out as previously described [69]. RNA was isolated from harvested cells or human tissues with Trizol reagent according to the manufacturer's instruction (Invitrogen, CA, USA). Real-time PCR reactions were performed using SYBR Premix DimerEraser (Takara, Dalian, China) and human GAPDH or U6 snRNA was used as an endogenous control for mRNA or miRNA detection, respectively. Expression of each gene was quantified by measuring Ct values and normalized using the 2^−ΔΔct^ method relative to U6 snRNA or GAPDH. The primers used in this study were depicted in supplementary Table 2.

**Table 2:** The Primers Of NEFL, Multidrug Resistance Factors And Stemness Facters

Gene name	Forward / Reverse primer(5′-3′)
NEFL	F: 5′-CTGGAAATCGAAGCATGCCG-3′ R: 5′-CGCCTTCCAAGAGTTTCCTGT-3′
ABCG2	F:5′-GAACCCAAGGAGATAGGAGA-3′ R:5′-CTAGACAGACTTCAACCAGG-3′
ABCC3	F:5′-CCTTCCAGGTAAAGCAAATG-3′ R:5′-GTGTCAGGGTAGAGTCCAAT-3′
ABCC5	F:5′-TTTTCAGGATGGCTGTATTCT-3′ R:5′-TGGCTTCTTTTCCAGTATGC-3′
ALDH1	F:5′-GTCCTACTCACCGATTTGAA-3′ R:5′-CTTGTATAATAGTCGCCCCC-3′
CD44	F:5′-CACAACAACACAAATGGCTG-3′ R:5′-CAATGCCTGATCCAGAAAAA-3′
CKIT	F:5′-TGGTATTTTTGTCCAGGAACT-3′ R:5′-GATTTGCTCTTTGTTGTTACCT-3′
KLF4	F:5′-AAGAGTTCCCATCTCAAGGC-3′ R:5′-GGTCATATCCACTGTCTGGG-3′
Nanog	F:5′-GAACTCTCCAACATCCTGAA-3′ R:5′-TATTCTTCGGCCAGTTGTTT-3′
Nestin	F:5′-CGGGCTACTGAAAAGTTCC-3′ R:5′-CTGAAAGCTGAGGGAAGTC-3′
SOX2	F:5′-TGGAAACTTTTGTCGGAGAC-3′ R:5′-CAGCGTGTACTTATCCTTCT-3′
GAPDH	F:5′-ATCAAGATCATTGCTCCTCCTGAG-3′ R:5′-CTGCTTGCTGATCCACATCTG-3′

### Immunoblotting

Cells were washed with ice-cold PBS buffer, scraped from the dishes, and centrifuged at 1200 rpm, 4°C for 15 min. Cell lysates were prepared using RIPA buffer supplemented with protease inhibitors (100 mM Tris (pH 7.4), 150 mM NaCl, 5 mM EDTA, 1% Triton X-100, 1% deoxycholate acid, 0.1% SDS, 2 mM phenylmethylsulfonyl fluoride, 1 mM sodium orthovanadate, 2 mM DTT, 2 mM leupeptin, 2mM pepstatin). The supernatants were collected and protein concentration was determined using BCA assay (Thermo, USA). Tumor tissues from human were grinded into powder in liquid nitrogen with RIPA buffer, and the total tissue proteins were extracted as described above. Aliquots of protein lysates were fractionated by SDS-PAGE, transferred to a PVDF membrane (Merck Millipore, Germany), and subjected to immunoblotting analysis according to the manufacturer's instruction. ECL Detection System (Merck Millipore, Germany) was used for signal detection.

### Luciferase reporter assay

The 3′-UTR of NEFL were synthesized and annealed, then inserted into the SpeI and HindIII sites of pMIR-reporter luciferase vector (Ambion) at downstream of the stop codon of the gene for luciferase. For its mutagenesis, the sequences complementary to the binding site of miR-381 in the 3′-UTR (NEFL:CTTGTAT) was replaced by TACTTGAC. These constructs were validated by sequencing. U251 cells were seeded into a 24-well plate for luciferase assay. After cultured overnight, cells were cotransfected with the wild-type or mutated plasmid, pRL-TK plasmid, and equal amounts of miR-381 or miR-NC. And the pRL-TK control vector was transfected as a control. Luciferase assays were performed 24 h after transfection using the Dual Luciferase Reporter Assay System (Promega, WI, USA). Firefly and Renilla reniformis luciferase activities were measured 24 h later. Experiments were performed in three independent replicates.

### Cell viability assay

Cell viability was determined by the CCK8 assay. Briefly, 2000 cells/well were seeded into 96-well plates and were treated by miRNA or plasmid vector transient transfection and/or TMZ (100μM) administration, and the absorptions of the cells were measured using a CCK8 kit (Beyotime Institute of Biotechnology, Jiangsu, China) according to the manufacturer's instruction at different indicated time points. Data were from three separate experiments with four replications each time.

### Matrigel chamber invasion assay

Invasion assay was determined using 24-well BD Matrigel invasion chambers (Corning Inc., Corning, NY) in accordance with the manufacturer's instructions. 2×10^4^ cells were seeded per well in the upper well of the invasion chamber in DMEM with 0.1% serum, the lower chamber well contained DMEM supplemented with 10% FBS to stimulate cell invasion. After incubation for 24 h, noninvading cells were removed from the top well with a cotton swab while the bottom cells were fixed with 4% paraformaldehyde, stained with 0.1% crystal violet, and photographed in three independent fields for each well. Three independent experiments were conducted in triplicate.

### Wound healing assay

Cells were cultured until reached 90% confluence in 6-well plates. Cell layers were scratched using a 10 μ L tip to form wounded gaps, washed with PBS twice and cultured. The wounded gaps were photographed at different time points and analyzed by measuring the distance of migrating cells from five different areas for each wound.

### *In Vitro* Chemosensitivity assay

The chemosensitivity of the cell was determined by the CCK8 assay. Briefly, cells were seeded and transfected with miRNA or Plasmid using the Lipofectamine 2000 (Invitrogen) transfection agent. After 12 h, the cells were reseeded in 96-well plate s at a density of 4,000 cells per well and treated with TMZ (12.5-800μM) for 48 h. Cell survival was analyzed using the CKK8 kit (Beyotime Institute of Biotechnology, Jiangsu, China) and absorbance was read at 450 nM on a microplate reader (Bioteck). Data were from three separate experiments with four replications each time.

### Statistical analysis

All experiments were performed three times and data were analyzed with GraphPad Prism 5 (La Jolla, CA, USA). The correlation between miR-381 expression and NEFL levels in tumor tissues were analyzed using Spearman's rank test. Differences between the variables of the groups were tested using the Student's t-test or one-way ANOVA, using the SPSS 15.0 program. A *p*-value of <0.05 was considered to indicate a statistically significant result.

## SUPPLEMENTARY MATERIAL FIGURE


