# Supplementary material for: Targeting miR-381-NEFL axis sensitizes glioblastoma cells to temozolomide by regulating stemness factors and multidrug resistance factors
Source: Oncotarget. 2014 Dec 18;6(5):3147–64. doi: 10.18632/oncotarget.3061 (PMC4413644; doi:10.18632/oncotarget.3061)
Supplement: Supplementary file 1 [file oncotarget-06-3147-s001.pdf]

Targeting miR-381-NEFL axis sensitizes glioblastoma cells to temozolomide by regulating stemness factors and multidrug resistance factors

Supplementary Material

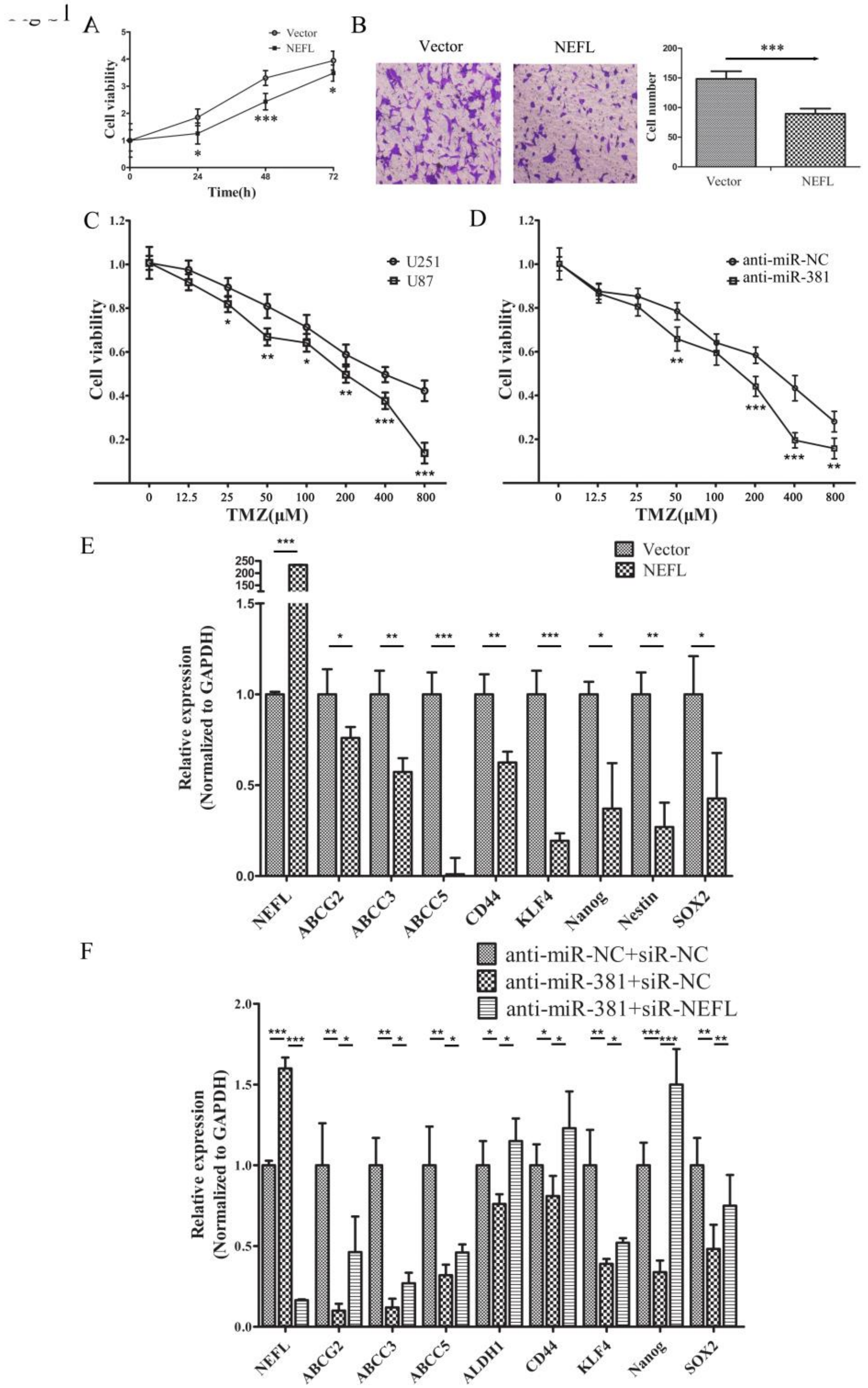

Figure S1: LNA-anti-miR-381 sensitizes U87 cells to TMZ by modulating multidrug resistance factors and stemness factors via NEFL

A: CCK8 assay showing the reduced proliferation of U87 cells transfected with NEFL.

B: Matrigel chamber invasion assay showing the reduced cellular invasion of U87 cells transfected with NEFL.

C: U251 and U87 cells were treated with various concentrations of TMZ for 48 h and then subjected to CCK8 assays. The U87 cells were less resistant to TMZ than the U251 cells.

D: U87 cells transfected with LNA-anti-miR-NC or LNA-anti-miR-381 were treated with various concentrations of TMZ for 48 h and then subjected to CCK8 assays. Inhibition of miR-381 increased the chemosensitivity of the cells to TMZ treatment.

E: qRT-PCR analysis showing that the expression of multidrug resistance factors (ABCG2, ABCC3, ABCC5) and stemness factors (CD44, KLF4, Nanog, Nestin, SOX2) was decreased in U87 cells overexpressing NEFL.

F: qRT-PCR analysis showing that the mRNA levels of multidrug resistance factors (ABCG2, ABCC3, ABCC5) and stemness factors (ALDH1, CD44, KLF4, Nanog, SOX2) were repressed in U87 cells after LNA-anti-miR-381 treatment and were restored by the NEFL-siRNA.

The data represent the mean±SDs of 3 replicates. * $p<0.05$; ** $p<0.01$; *** $p<0.001$.
